# The Impact Factor of Balkan Medical Journal Continues to Rise

**DOI:** 10.4274/balkanmedj.galenos.2020.2020.5.001

**Published:** 2020-08-11

**Authors:** Zafer Koçak

**Affiliations:** 1 Department of Radiation Oncology, Trakya University School of Medicine, Edirne, Turkey

According to the 2020 Journal Citation Report by Clarivate Analytics, the impact factor of Balkan Medical Journal arose by 1.53 in 2019, which reflects 27% increase compared to last year. It has become one of the most prestigious journals in the Balkans and Turkey because of this new score. Based on this development, I would like to talk about what has been done in the last 4 years on behalf of the editorial team.

After taking over as editor-in-chief in autumn 2016, for the first time in the history of the journal, an editorial independence agreement was signed between the editor and the owner of the journal. However, in the early days of this new appointment, the editorial team faced a publisher change. The publishing house change which is one of the most difficult processes and changes of a journal was successfully realized with the support of the Dean and the intensive work of the editorial board. From 2017, we decided to continue with the Galenos publishing house. In November 2016, the news that the journal was accepted to be indexed in the PubMed/MedLine increased our motivation. Therefore, as stated in the history of the journal (1), the first four months of our editorial task were very challenging.

Another innovation in the year 2017 was the journal cover. Founded in 1488, Sultan Bayezid II complex of Edirne is the first example of a centrally planned medical center and considered the pioneer of modern hospitals thus, the roots of medical education in the city date back to the founding day of the school. The third issue therefore was the change to a new journal cover, designed to symbolize the silhouette and historical heritage of the Bayezid II Hospital (2).

In the last quarter of 2017, we signed one of the most challenging changes. After working hard with our publisher, we created a new and improved website that would better serve readers, reviewers, and authors (3). This dynamic website would contribute to the visibility and recognition of the journal. Another innovation in that same year was the articles and content of the magazine became visible on the social media.

In the third quarter of 2018, the editorial team of the Balkan Medical Journal was pleased to announce the new policy (gender and gender reporting) of the Balkan Medical Journal (4). We have chosen to be a part of global efforts to report and evaluate gender and gender differences in scientific research. With this policy, we started to encourage authors to use the terms gender (to report biological factors) and gender (to report identity, psychosocial or cultural factors) correctly.

One of the most challenging changes in 2018 was the switch to the online submission system. In the second half of 2018, as a result of the depreciation of the Turkish lira, we had to reduce the cost of the journal. After a long-term search by the editorial team and the publisher, we decided that the Manuscript Manager peer review system would meet all our needs (5). With this decision, starting from the last week of November 2018, we were able to use the new system. We have successfully overcome this transition period.

Another job waiting to be done for us was the journal’s legal regulation. The editorial independence agreement between the owner of the journal and the editor alone did not guarantee the institutional independence of the journal therefore, a legal regulation was needed. We thought that the implementation of the journal legal regulation would guarantee the independent and responsible management of the journal, protect the freedom of publication, and ensure the transparency of the team at work. In addition, the effort to maintain the editorial freedom had posed distinct challenges arising from regional dynamics in the Balkan region. After working closely with the Dean’s Office and rectorate’s legal commission, the Balkan Medical Journal Legal Regulation came into force in January 2019 (6).

As a member of IJMCE, in January 2019, Balkan Medical Journal declared compliance with the ICMJE data sharing statement (7). As a journal of this geography, it should not be a surprise to say that we have difficulties in implementing this policy. It is obvious that we need more time in this regard. One of the most important factors in the implementation of this policy is the willingness of the authors and institutions to share their data.

In the second quarter of 2020, the whole world was shocked by the new coronavirus pandemic. The COVID-19 outbreak pressured journals and authors to quickly publish many academic articles about the new coronavirus. This edition led to some editorial practices that ignore ethical principles. During this period, we witnessed many qualified journals withdrawing or retracting COVID-19 articles. This period has shown how important the competence of editorial board members is (8). In addition, this pandemic process has become a real opportunity for predatory journals (9). As the editorial board, I think we did a good job in this period. In all the original articles on COVID-19 that were not rejected by the Editor-in-Chief but sent to the relevant editor, they were either rejected or withdrawn after the peer review or detailed and rigorous evaluation. In the accepted articles (Invited review, case report and letter to the editor), there were extensive revisions of the relevant editor and/or editor-in-chief.

Table 1 summarizes our struggle in the past 4 years. The upward trend in the journal's impact factor stemmed from the rigorous selection of articles, good communication with readers and authors, the application of ethical standards, and the devoted work of the editorial board and the publisher. We believe that the key to being a second quarter journal is the continuity of these policies.

## Figures and Tables

**Table 1:**
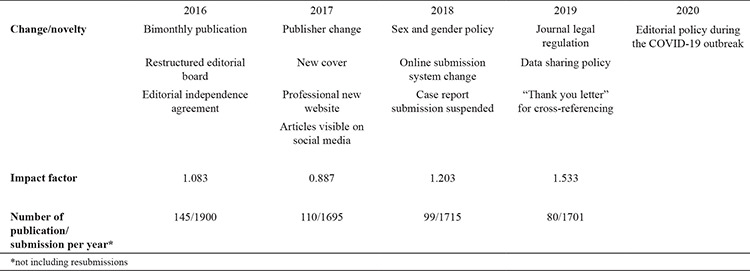
Summary of the changes/novelties made in the last 4 years by the editorial board and the change of impact factor and submission numbers by years
